# Antiretroviral Drug-Resistance Mutations on the *Gag* Gene: Mutation Dynamics during Analytic Treatment Interruption among Individuals Experiencing Virologic Failure

**DOI:** 10.3390/pathogens11050534

**Published:** 2022-05-03

**Authors:** James R. Hunter, Domingos E. Matos dos Santos, Patricia Munerato, Luiz Mario Janini, Adauto Castelo, Maria Cecilia Sucupira, Hong-Ha M. Truong, Ricardo Sobhie Diaz

**Affiliations:** 1 Department of Medicine, Federal University of São Paulo, Sao Paulo 04039-032, Brazil; jhunter@unifesp.br (J.R.H.); domingos.matos@yahoo.com.br (D.E.M.d.S.); patricia.munerato@cepheid.com (P.M.); janini@unifesp.br (L.M.J.); adauto.castelo@unifesp.br (A.C.); cecilia.araripe@unifesp.br (M.C.S.); 2 Department of Medicine, University of California, San Francisco, CA 94158, USA; hong-ha.truong@ucsf.edu

**Keywords:** antiretroviral virologic failure, antiretroviral resistance, analytical treatment interruption, fitness cost, *gag* gene

## Abstract

We describe drug-resistance mutation dynamics of the *gag* gene among individuals under antiretroviral virologic failure who underwent analytical treatment interruption (ATI). These mutations occur in and around the cleavage sites that form the particles that become the mature HIV-1 virus. The study involved a 12-week interruption in antiretroviral therapy (ART) and sequencing of the *gag* gene in 38 individuals experiencing virologic failure and harboring triple-class resistant HIV strains. Regions of the *gag* gene surrounding the NC-p2 and p1-p6 cleavage sites were sequenced at baseline before ATI and after 12 weeks from plasma HIV RNA using population-based Sanger sequencing. Fourteen of the sixteen patients sequenced presented at least one mutation in the *gag* gene at baseline, with an average of 4.93 mutations per patient. All the mutations had reverted to the wild type by the end of the study. Mutations in the *gag* gene complement mutations in the *pol* gene to restore HIV fitness. Those mutations around cleavage sites and within substrates contribute to protease inhibitor resistance and difficulty in re-establishing effective virologic suppression. ART interruption in the presence of antiretroviral resistant HIV strains was used here as a practical measure for more adapted HIV profiles in the absence of ART selective pressure.

## 1. Introduction

In the late 1990s and the early years of this century, researchers studied the clinical benefit that analytical treatment interruption (ATI) might be able to offer HIV-1 patients experiencing antiretroviral virologic failure given the limited options of salvage therapy available at that time. At that time, treatment interruption was considered as a therapeutic option. However, these studies led to the conclusion that complete ATI cannot provide clinical benefit to justify its use as an adjunct to antiretroviral therapy (ART) programs [1,2,3,4,5]. Studies of alternate ATI schemes involving shorter cycles of treatment interruption and ART treatment also proved nonviable [6,7,8]. As a result, clinicians and authorities stopped using ATI as a therapeutic tool.

The data were gathered for this study toward the end of the period in which ATI was considered an option, as researchers believed that ATI would hasten the reappearance of wild-type virus. In turn, wild-type virus would facilitate the subsequent reintroduction of ART [9,10,11,12]. The impact of ATI on other genes that are directly related to the selective pressure of antiretrovirals in use has hardly ever been evaluated [13,14]. The present analysis exploits the withdrawal of antiretroviral selective pressure on the *gag* gene. A companion paper explores the mutation dynamics and drug resistance on the *pol* gene in the regions that produce the protease and reverse transcriptase enzymes [15].

### 1.1. Inverse Substrate–Protease Examination

This paper is the first to use these data to examine systematically the mutation dynamics of viral resistance related to ATI on the *gag* gene. Our study used an inverse substrate–protease method. This differs from the usual approach in which examination of emerging mutations in the *gag* gene occurs after a mutation in the protease region of the *pol* gene is identified. Instead, we examined mutations in the *gag* protein in the presence of selective pressure by the protease inhibitor and then examined how it related to resistance mutations in the *pol* protease region. This strategy provided insights into the *gag* mutations that might relate to resistance to protease inhibitors, either increasing the protease inhibitors’ resistance or restoring HIV fitness related to protease mutations.

We examined both the *gag* cleavage sites as well as the peptides on the *gagpol* polyproteins that compose new virions. The formation of virions relies on the translation of the *gagpol* polyprotein and the cleavage of this protein in an ordered way to transform the structure from an ill-defined doughnut inside the envelope into a clearly defined matrix (MA) layer outside the capsid cover (CA) for the viral RNA coupled with the nucleocapsid (NC). Protease acts as the scissors to cut the segments of the polyprotein into the correct segments. These cuts must occur in the correct sequence for the virion to mature to the correct final form.

**Figure 1:**
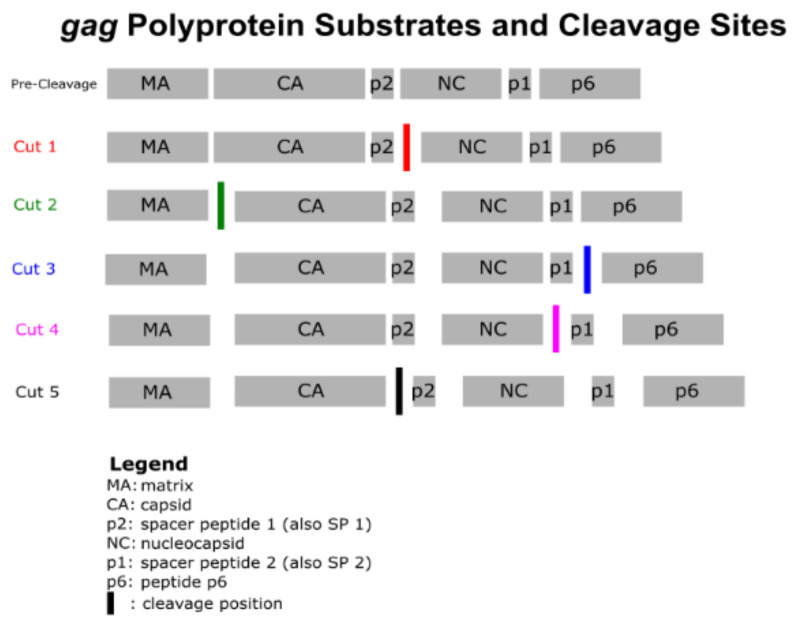
*Gag* polyprotein substrates and cleavage sites.

Figure 1 shows a schematic representation of the *gag* portion of the *gagpol* polyprotein with its component peptides and cleavage sites [16,17]. In this study, we used as a reference genome the strain known as SF-2, which is considered the HIV clade B North American consensus sequence [18]. Reference locations were taken from SF-2. Table 1 specifies the cleavage site locations by codons for the SF-2 genome and the residues that are located there.

In addition to the principal components of the precursor polyprotein that protease cleaves, there are two spacer peptides, p2 (also known as SP1) and p1 (also known as SP2). While p2 appears to have a role in maturation of the virion, the function of the p1 spacer is not known. In the reassembly of the virion into a functional virus, the spacer peptides are trimmed and not used. There is disagreement among studies as to whether it is the sequence of the *gag* component that the protease recognizes and acts on [16] against a view that there are secondary structural aspects of the peptide, particularly the three-dimensional conformation of the substrate [19], that trigger the action of the protease.

### 1.2. Viral Resistance and the gag Gene

Viral resistance to protease inhibitors (PIs) stems not only from mutations at the protease region of the *pol* gene. Resistance has also been shown to result from mutations of the *gag* gene in those regions that build the natural substrates of new virions [14]. PIs also play a role in suppressing the maturation of virions, thereby preventing the restructuring of the virion that permits it to function as a mature virus capable of infecting new cells [16]. Mutations in these substrates can make the substrate invisible to the PI and therefore resistant. This allows the cleavage to occur unimpeded. The literature cited here states that resistance occurs primarily at the cleavage sites themselves. However, as we will show, resistance mutations can develop as well within the substrates, while the cleavage sites themselves remain wild-type.

Once infected, the patient’s HIV-1 begins to evolve and continues to do so even as the virus faces long-term replication suppression due to ART [20,21]. Given that ART suppresses the susceptible HIV strains, continuous selection of ART-resistant strains will lead to the emergence of mutational strains that have greater replication fitness [22]. These strains permit the viral load of the patient to increase, which, in turn, leads to greater inflammation and transmission opportunity.

Additionally, some anatomical sites such as the central nervous system and genital tract may not be readily accessible to some classes of antiretroviral drugs. For example, the brain and genital tract absorb protease inhibitors poorly [23]. These not easily accessible compartments, along with resting CD4^+^ lymphocytes, create HIV-1-resistance havens and permit movement of the virus between compartments with measurable resistance levels [24,25].

Viral fitness, the ability to create adapted and replicating infectious progeny, is inversely related to the number of incorporated antiretroviral resistance-related mutations [26,27,28]. In the absence of the selective pressure exerted by antiretroviral drugs, the wild-type virus or a less resistant one has a greater capacity to outgrow the resistant strains [29,30,31].

This general resistance fitness adaptation applies to the *gag* gene as well as the more traditionally studied *pol* regions that produce the three enzymes needed for viral replication: reverse transcriptase, protease and integrase. Existing studies of the contribution of *gag* to resistance to PIs characterize the mutations originating in this gene, particularly at the NC/p1 cleavage site, as “compensatory”; that is, they restore the activity and replicative capacity of the mutated protease as it acts on the substrate in this gene [32]. There are a small number of studies that assert that *gag* mutations function as primary rather than secondary resistance mechanisms [33]. Almost all these studies are confirmatory in nature [34]. They refer back to the studies already carried out that focused on the cleavage sites in choosing the regions of *gag* to sequence. We have included a wider region in this study to test the idea that mutations directly affecting the substrate components might also become primary points of resistance.

### 1.3. Objectives

The current study aimed to describe *gag* evolution from plasma RNA throughout the 12 weeks of ATI. We examined resistance mutations at the *gag* cleavage sites which have frequently been observed among individuals failing PI-containing regimens. Additionally, we examined mutations in the substrate sequences themselves. We were seeking cases of codons with mutations pre-ATI that would become wild-type again at the end of the interruption. We used this result as the basis for looking backward to mutations in these patients to find which PI-resistance mutations the *gag* mutations relate to and determine whether these mutations follow the same dynamic of return to the wild type.

## 2. Results

### 2.1. Patients

This study included 36 patients with HIV-1 triple-class antiretroviral resistance mutations and 2 patients with chronic HIV-1 infection but no detectable HIV-1 resistance-related mutations. This latter group served as controls. There were one male and one female control subjects. The mean age of the controls was 37.0 years (standard deviation of 1.41 years). The patients harboring resistance included 28 males (77.8%) and 8 females (22.2%). The mean age of the resistant group was 38.75 years (standard deviation of 7.77 years).

### 2.2. Gag Sequences from Patients

Overall, 33 of the 36 patients with resistant HIV strains had their gag virus genes sequenced pre- and post-ATI for a total of 66 sequences. These sequences ranged from 332 to 513 codons, with a mean of 445.8 codons per sequence (95% confidence interval of 439.0 to 452.5 codons). When aligned to reference sequence SF-2, the sequences had an average length of 506.9 codons (95% confidence interval of 503.1 to 510.7 codons and a range of 502 to 563 codons). This range includes the matrix, capsid, p2 spacer, nucleocapsid, p1 spacer and p6 regions of *gag*.

Among the sequences taken at baseline (pre-ATI), the translation software could not successfully assign an amino acid to a mean of 22.0 (4.3%) of the codons per patient. For the post-ATI sequences, the loss was a mean of 21.1 (4.2%) of the codons per patient. Of the 1155 codons sequenced for the cleavage sites shown in Table 1 (35 codons per patient for 33 patients), the pre-ATI loss either to uninterpretable translation or to an alignment gap was 58 individual codons (5.1%). For the post-ATI sequences, this loss increased to 118 (10.2%).

### 2.3. Mutations Pre- and Post-ATI for the Entire Gag Gene

**Figure 2:**
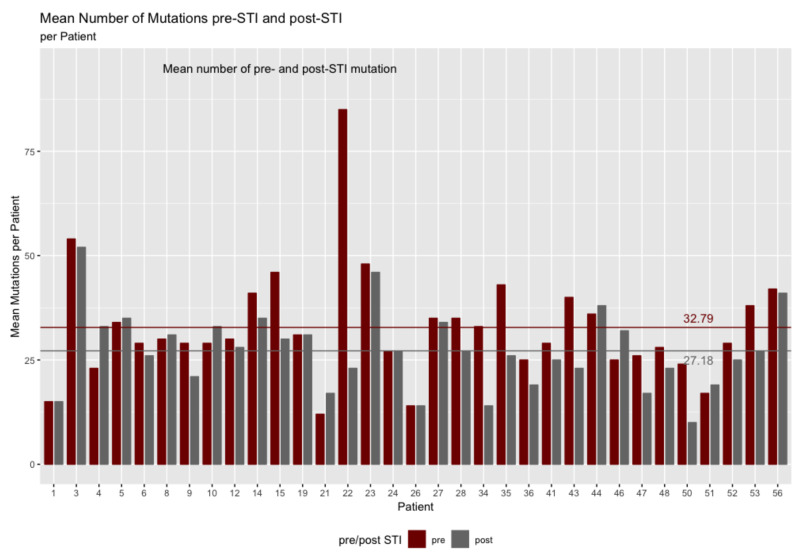
Number of mutations in the *gag* gene pre- and post-ATI per patient.

The patients’ viruses mutated across all regions of the gag gene. According to the North American consensus sequence pre-ATI, the patients averaged 32.8 codons with mutations (6.5% of the codons). The number of mutations per patient declined post-ATI to 27.2 (5.3%). Figure 2 shows the number of mutations pre- and post-ATI for all patients. The median number of mutations post-ATI is 27 compared to 30 pre-ATI.

The difference between the pre-ATI number of mutations per patient and the post-ATI number is close to statistically significant according to a Welch’s two-sample *t*-test (α = 0.05, t = 1.97, df = 57.2, *p* = 0.054).

Also supporting the hypothesis that mutations tend to return to the wild type after a treatment interruption is a view of how many patients had fewer mutations than before the interruption: 21 patients had reductions in the number of mutations and 12 patients increased the number of mutations after the interruption. Patients showed an average reduction in the number of mutated codons of 5.6 codons per patient (mutated codons pre-ATI less mutated codons post-ATI). The 95% confidence interval for this difference varies between 1.2 and 10.0 codons per patient. This suggests that a return to the wild type in the gag protein does accompany an ATI.

### 2.4. Mutations in the Regions of Gag

**Figure 3:**
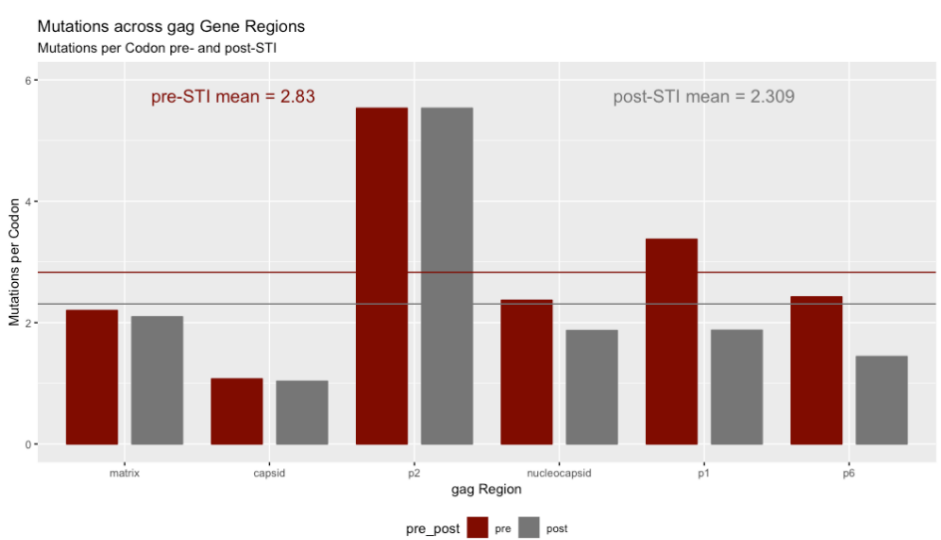
Mutations per codon across *gag* regions.

The patients’ viruses mutated across all regions of the gag gene. Table 2 and Figure 3 show the number of mutations pre- and post-ATI for all six *gag* regions. The analysis is corrected for the size of the region, since they range from 15 codons in spacer p2 to 231 codons defining the capsid substrate. The measure then becomes mutations per codon to regularize them.

The graph shows that the smallest region, p2, had substantially more mutations per codon than the much larger capsid or matrix regions. p2 had 5.53 mutations per codon, in contrast with 2.20 for the matrix substrate and 1.07 for the capsid. In raw counts of mutations, the matrix substrate had 576 mutations across its 134 codons and the capsid had 576 mutations on its 231 codons. These counts correct for the losses for uninterpretable codons and those occurring in gaps.

As with the analysis on a per patient basis, the regions show a small mean return to the wild type. However, this return is marginally not significant at α = 0.05 using a Kruskal–Wallis rank sum test of differences among the six regions as a group. The chi-square value of the test was 10.498 with 5 degrees of freedom and resulted in a *p*-value of 0.0623.

### 2.5. Gag Cleavage Site Mutations Pre- and Post-ATI

**Figure 4:**
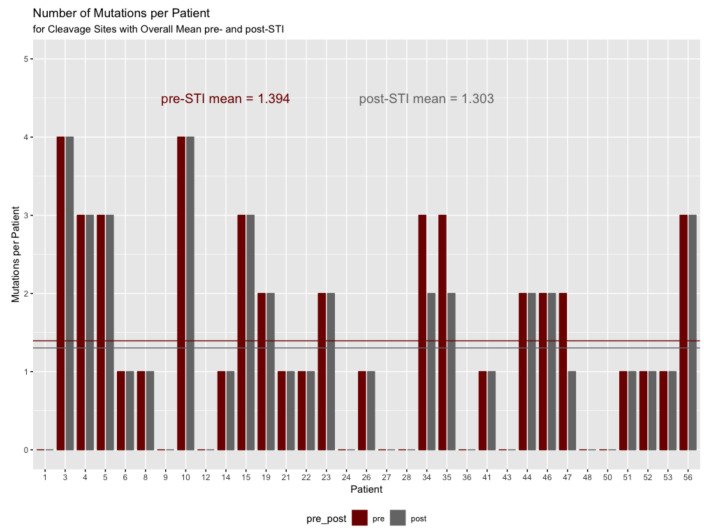
Mean mutations for cleavage sites.

Focusing on the codons within the five cleavage sites, we found that a mean of 1.39 codons had mutated pre-ATI (4.0% of the 35 codons in the cleavage sites). Post-ATI, this total had declined to 1.30 (3.7%). As Figure 4 shows, there was a decline of 0.09 codons per patient, which did not represent a significant difference (by a small margin) on a paired value *t*-test (α = 0.05, t = −1.7889, df = 32, *p* = 0.0831).

When focused on the cleavage sites between pre- and post-ATI, the differences raise doubts as to whether a clear picture emerges. Three patients (9%) showed a decline in the number of mutations and 20 (61%) showed no change in the number of mutations at the cleavage site locations. It should be noted that 10 patients (30%) showed no mutations either before or after ATI in the cleavage sites.

**Figure 5:**
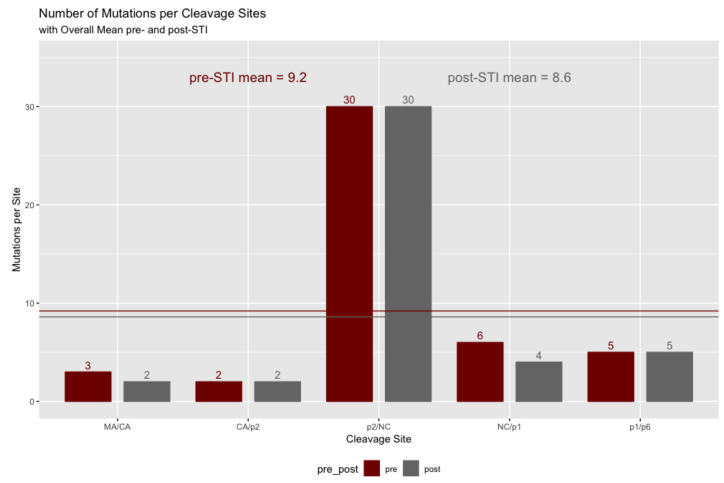
Mean mutations for cleavage sites.

Among the cleavage sites, the junction between the first spacer peptide (p2) and the nucleocapsid (NC) contained the greatest focus of mutations. Thirty codons (both pre- and post- ATI) in total across the p2/NC region (codons 377–383) were mutated in our sample. This was also the case for the p2 region, as noted in Table 2. Figure 1 shows that p2/NC is the first site that protease cleaves to separate the nucleocapsid from the spacer peptide p2. Its complete liberation from the substrate comes after the fourth cleaving. The next highest number was six mutations in the NC/p1 region, pre-ATI. As can be seen in Figure 5, there was little decline between the pre- and post-measurements of ATI. Only the MA/CA showed a single decline and NC/p1 a decline of two mutations. The pre-ATI mean was 9.2 mutations across the sites, declining to 8.6 mutations at the end of the study.

Among the mutations recorded at cleavage sites, only four returned to the wild type, lower than our expectation. Table 3 includes these codons and the patients involved, along with residues and the cleavage site locations. Also in Table 3, we examined the patterns of mutation within the five cleavage site regions. In both the MA/CA and CA/2 regions, the mutations were focused on a single codon (134 for MA/CA and 364 for CA/p2). In both cases, the codon mutated was located before the actual center of the cleavage.

For the p2/NC site, the two codons furthest from the actual cutting point between 434 and 435 were the codons most frequently mutated. Codons 431 (mutated in 17 patients) and 432 (mutated in 7 patients) account for 80.0% of the mutations in the cleavage site. It is also important to note that in none of the codons was the p2/NC cleavage site returned to the wild type, despite this being by far the site with the greatest number of mutations in our sample. In the last two cleavage zones, NC/p1 and p1/p6, no clear pattern emerges about the mutation of the codons themselves.

### 2.6. Gag Mutations in Plasma

Thus far, we have focused on the structure of the mutations in the gag gene, identifying the genomic characteristics that relate to their location on the substrates that the coding of gag defines. This is the key to the inverse substrate–protease analysis we have followed. We have also identified mutated protease residues in the pol gene as discussed above.

Table 4 relates the mutated protease residues to the mutated gag residues. Here, we identified 50 sequences in which we had data on both the gag substrates and the protease region of pol. This covers 24 unique codons. Codon 455 had two variants, one from patient 28 and one from patient 43. This codon lies two codons downstream from the p1/p6 cleavage site. The PI mutations are limited to those considered by the IAS panel [35] to warrant “particular caution” regarding the use of associated drugs.

Certain patterns emerge. Some of the mutations affect codons that are important to the functioning of protease and some are simple polymorphisms that do not appear to have a role in PI resistance.

The table contains mutations that reverted to the wild type at the end of the 12-week ATI. We have classified the reverting mutated codons by *gag* codon number and listed the baseline and endpoint amino acid for each patient. For example, codon 76 in the matrix region of the *gag* polypeptide was only mutated in patient 43. At baseline, this patient had a lysine (K). This returned to the wild-type arginine (R) in the final sequence acquired. At the beginning of the study, the sequencing of the protease region of the *pol* gene had a single residue with mutations (L90M). The mutation disappeared at the end of the study

Of the 25 overall mutated gag mutations that returned to the wild type, only five did not lose all their protease mutations. Three of these five, patient 6 at codon 79 and patient 34 at codons 111 and 134, retained the L90M mutation they carried at baseline. Patient 48 at both codons 121 and 122 retained an I84V protease mutation as well as the L90M. It is also important to note that all these cases are in the matrix region and only the mutation at codon 134 (Y134F) is located at a cleavage site.

We also repeated this analysis with a looser selection criterion. Instead of requiring that the baseline mutation return to the wild type at the end of the study, the mutation merely had to change to any other amino acid. We wanted to capture those cases where mutations can pass through intermediate changes before returning to the wild type. In this case, we captured 99 sequences pre- and post-ATI. We have included these data in Supplementary Table S1. The results were similar to those of Table 4. We found with this criterion that 71% of the mutated *gag* sequences were associated with protease sequences that returned to the wild type instead of 80% with the Table 4. Again, the matrix substrate was the dominant region in the sample and six sequences were located in cleavage sites.

### 2.7. Minor Protease Mutations in Relation to Gag Mutations

We also examined the minor IAS mutations associated with the *gag* mutations that reverted to the wild type (see Supplementary Table S2). The most prevalent of these minor mutations is L63P, which appears in 19 of the sequences and persists post-ATI in 13. However, codon 10 of the protease peptide has the greatest frequency of mutation. Instead of the standard leucine (L), five patients have a valine (V) substitution and 17 an isoleucine (I) substitution, with two more being classified as L10I/V. For 12 of the 24 total L10 mutations pre-ATI, the mutation was lost at the end of the study. None of the valine substitutions returned to the wild type. Six of the pre-ATI L10I mutations were associated with mutations in the p6 region of *gag*, while all but one of the L10V mutations were associated with mutations in the matrix substrate.

## 3. Discussion

The sudden withdrawal of the selective pressure of antiretrovirals causes an enormous disequilibrium in the relationship of HIV to its environment, i.e., the human body. For instance, it is known that HIV evolution for conserved regions such as the gag gene is less than 1% per year [36]. Upon ATI, we have observed here a much higher and faster change in the HIV genetic profile at the *gag* gene. The sudden change in the HIV quasispecies profile caused by this sort of ATI may sometimes lead to the reemergence of ancient HIV virion profiles, even in distant genes that are unrelated to the selective pressure exerted by antiretrovirals at the reverse transcriptase and protease regions of the *pol* gene. As an example, as more “primitive” wild-type HIV strains replace the more contemporary antiretroviral resistant strains upon ATI, R5 strains also reemerge as predominant strains, replacing the CXCR4 tropic strains that naturally evolved over time, with no immediate chance left for virus recombination. [14].

Another issue of extreme interest is the role that mutations not related to the inhibited enzyme may have in antiretroviral resistance. For instance, it has been recently demonstrated that mutations in the *nef* gene at the 3′ polypurine tract region are selected and cause resistance to integrase inhibitors. [37]. It has also been elegantly demonstrated that mutations at the protease cleavage sites are not only related to HIV fitness restoration upon emergence of selected mutations at the protease region of the *pol* gene but also cause resistance to protease inhibitors themselves [16,17].

Therefore, determination of resistance-related mutations or mutations that emerge to restore the fitness loss caused by resistant-related mutations may be also determined not only by the HIV evolution vis-à-vis virus replication in the presence of inhibitors but also by the change of HIV profile upon interruption of antiretroviral selective pressures. To investigate the *gag* gene, we used the inverse substrate–protease examination strategy.

### 3.1. Process of Reversion to the Wild Type

As we stated in the companion paper, the picture for reversion to the wild type is not very clear whether dealing with mutations on *pol* or on *gag*. In contrast with other studies, this study shows that return to the wild type is not a given after ATI [11,31]. Here, the number of mutations per patient declined from 32.8 codons to 27.2, a reduction of 17.1%—well below our results for the *pol* gene, where 87.5% of the codons studied in plasma returned to the wild type [1]. Analyzing the reduction in the number of codons with mutations directly within the cleavage sites, the decline in the mean number of mutations per patient of 0.09 codons (reduction of 6.5%), while nearly significant, was very slight.

As we show in Table 2, Table 3 and Table 4, mutations disappear, each at its own rate. There appears to be little or no uniformity or predictability in the rate of reversion. Theoretically, once the selective pressure of ART is withdrawn, the wild strain should reemerge rapidly. That they do not suggests that some proviral compartments last longer than we have seen previously. This is especially so since viruses with distinct resistance-related mutation profiles may reemerge from latency once ART is withdrawn or due to the differential replicative capacity of distinct strains in each HIV quasispecies. When single-genome amplification of antiretroviral resistant strains is performed, mutations that are detected in the population sequences may not be present in all HIV strains but be dispersed among distinct viruses of the HIV quasispecies at a given moment [38]. We must also consider that for experienced patients with virologic failure to multiple HIV regimens, ancient HIV strains with still different mutation profiles may exist in individual cells or groups of cells. Here, we describe a set of mutations determined by inverse substrate–protease analysis that possibly relate to PI resistance: K103Q, S111C, K114R, A121D, A122T, Y134F (matrix); I149L, V217E, T243S (capsid); T375N (p2); R386K, K390R, R420K (nucleocapsid); A435V (p1); L451P, P455L/T, P457L, F469L, T472A, T476I (p6).

### 3.2. Cleavage Sites as the Foci of Gag Mutation Analysis

Previous research has identified the most frequently observed cleavage site mutations as A433V (NC/p1 site), L451F (p1/p6 site) and K438R and I439V, both downstream of the NC/p1 site [31]. Of these mutations, only the mutation at codon 451 appears in our sample. Since both the 431 and 437 mutations are missing from our sample and both showed a high degree of resistance to PIs, the current study is unable to evaluate the previous claim that they are central to PI resistance [16]

This previous research tends to put mutations to the substrate regions into a secondary role in determining resistance based on the logic that the cleavage sites are the locations that the protease will cut and are therefore most vulnerable to PIs during ART and liberated from the pressure of ART during treatment interruption. To the extent that previous studies have recognized mutations to the substrate regions of *gag*, it has been to assert that, during ART, PIs will suppress the cleaving activity at the cleavage sites and therefore favor the substrate as the location for the formation of resistance mutations [16].

Our finding that very few of the mutations we studied that returned to the wild type were in the cleavage sites suggests that perhaps they have no greater importance than the substrates whose extent they define. While the mechanism by which PIs may not stop cleavage due to failure to recognize a mutated cleavage site is clear, a future line of research should seek to understand more thoroughly how mutations to the substrate peptides themselves can deceive the PIs, thus allowing them to continue their maturation. This could help define a broader role for *gag* mutations and positions related to fitness restoration or increased resistance to PIs in the presence of PI resistance.

### 3.3. Relation of Gag Mutations to Protease Inhibitor Resistance

Through the inverse substrate–protease approach, we determined which protease mutations occurred in the same patient as the *gag* substrate mutations we keyed them to. The relationship between mutations of *gag*, their reversion to the wild type and protease mutations on *pol* suggests that larger samples are necessary to pick apart the underlying biological logic of drug resistance to PIs and the role of gag mutations in directing the formation of new virions and furnishing the components that will compose the virion.

We observed the persistence of the L90M protease mutation in patients who had a variety of *gag* mutations that reverted to the wild type. This profile (PI mutation associated with the *gag* wild type) may represent the backward evolution of acquired PI resistance, which is from the wild type to the PI mutation (step one) followed by the PI mutation associated with the *gag* mutation (step 2). If this is true, a larger follow-up would be needed to ascertain that the PI mutation would next be replaced by wild-type strains. One can also speculate that the *gag* mutations that reverted to the wild type during ATI were more closely related to PI resistance than to fitness restoration in the presence of the PI mutation.

The role of the L10 protease mutations also suggests that the behavior of protease and its action to cause the maturation of new virions is more complex that previously recognized [39]. It is interesting to note that in 5 out of the 25 different patient/codon combinations in Table 4, L10I was the only mutation still present post-ATI, suggesting that, although selected for PI and contributing to PI resistance, these mutations do not strongly affect virus fitness. The same occurred for L63P, as can be seen in Supplementary Table S2, which suggests that it does not have an important role in viral fitness despite its frequency.

### 3.4. Impact of NGS on Mutation Persistence Analysis

We recognize that a more detailed analysis of HIV quasispecies in this study would greatly benefit from single-genome amplification or next-generation sequencing tools. It has recently been demonstrated using next-generation sequencing (NGS) that transmitted drug-resistance mutations representing small percentages of the viral population do not persist during infection because they are negatively selected in the first year after HIV-1 seroconversion [40].

We recognize that more intensive cell population evaluations, additional immunologic markers or direct HIV fitness evaluations could provide still greater insights into the mechanisms of viral dynamics after ATI among patients experiencing virologic antiretroviral failure. However, the number of patients evaluated here is larger than those described in other similar studies. We have therefore been able to show how the reversion of the virus to a wild type after ATI among individuals failing antiretroviral therapy is not a clear and consistent process across the full range of *gag* and *pol* codons studied and provide insight into the persistence of distinct resistance-related mutations selected by distinct antiretroviral classes.

Third, we believe the study could have benefitted from additional control participants to make comparisons between the controls and the resistant participants clearer. However, given the gap between the sampling of the data and its analysis in this study, that was not possible.

## 4. Materials and Methods

### 4.1. Patients

Thirty-six HIV-1-experienced treatment patients who were treated at the Federal University of Sao Paulo, Brazil, in 1999 agreed to participate in the study on ATI. The samples were collected from June 1999 to June 2000. All patients were followed for 12 weeks. The total sample size of 38 was large for studies of this type at the time that it was carried out. A non-systematic review of 13 studies on treatment interruption that paralleled this study in intent and method published between 1999 and 2004 had a median number of participants of 24 and an interquartile range of 28 (10–38). Of the studies reviewed, only 4 had more or the same number of participants as the present study. (See Supplementary Table S3.)

Eligible male and female patients met the following criteria: 18 years or older; experienced multiple ART failures while using nucleoside analog reverse transcriptase inhibitors (NRTIs), non-nucleoside analog reverse transcriptase inhibitors (NNRTIs) and protease inhibitors (PIs); had detectable viral loads above 5000 copies/mL; showed no signs of AIDS. All had used the three ART classes and had shown triple-class antiretroviral virologic failure. All participants had signed an informed consent form and the study was approved by the Ethical Review Board at the Federal University of Sao Paulo, Brazil (number 0598/03).

Previous use of antiretrovirals before the experimental period revealed a variety of the antiretrovirals then available. Supplementary Table S4 shows how many patients used each antiretroviral. All the patients except one were compatible with subtype B of the HIV-1 virus. The discordant individual had a B/F subtype on the *gag* gene. [14]

### 4.2. Design

All patients enrolled on ATI at day zero started a prophylactic regimen against opportunistic agents as needed (data on file). CD4+ T cell counts were taken at 15-day intervals, viral loads were performed monthly and two plasma genotyping tests were performed at baseline and week 12 from the *gag* gene. No patient presented AIDS defining conditions during the study period.

### 4.3. Sequencing of the HIV-1 Gag Gene

For the analysis of genotypic resistance of plasma, RNA was purified and reverse transcribed as previously described [14]. The proviral DNA and plasma cDNA was submitted to a nested-PCR reaction as previously described for *gag* [41] and *pol* fragments [42].

For the reverse transcriptase and protease regions, the nested-PCR fragments were purified using a PCR Purification kit (QIAGEN Inc., Chatsworth, CA, USA) and sequenced using a Thermo Sequenase fluorescent-labeled primer cycle sequencing kit—RPN 2436 (Amersham Pharmacia Biotech UK Limited, Buckinghamshire, England). Primers were labelled by CY5.5. The products of the sequence reaction were analyzed using a DNA—Long Reader Tower (Visible Genetics Inc., Toronto, ON, Canada). Base-calling was performed using GeneObjects software (Visible Genetics Inc., Version 3.0, 1998, Toronto, ON, Canada) and the bases were aligned and assembled using GeneLibrarian software (Visible Genetics Inc., Toronto, ON, Canada). For the *gag* gene, sequencing was performed in an ABI 3100 Genetic Analyzer (Applied Biosystems—Waltham, MA, USA).

The resulting sequences for each sample were interpreted using the 2017 edition of the IAS–USA drug-resistance mutations list [35].

### 4.4. Inverse Substrate–Protease Analysis

We developed the inverse substrate–protease method of examining the relation of mutations to codons in the gag substrate polypeptide to mutations to the protease enzyme itself.

This approach has three phases. First, we analyzed which codons showed a change between baseline and 12 weeks. This can produce one of three results: no change during the ATI, change back to the wild type, as defined by the SF-2 reference sequence, or any other change. Second, we verified which mutations came closest to the consensus sequence at 12 weeks. Third, we associated those gag codons with the PI-resistance mutations on *pol* that appear in the IAS mutation list.

### 4.5. Quantitative Analysis

All data analysis was conducted using the R Statistical Computing System on a Macintosh computer (version 3.5.0) [43]. Each patient’s *gag* gene was sequenced at the beginning of the study, pre-ATI, and again at the end of the study, post-ATI. We then translated these sequences to amino acids using R packages from the Bioconductor project [44] and the package seqinr [45]. We aligned the pre-ATI and post-ATI sequences with the SF-2 reference genome using the Bioconductor package msa [46]. We used the CLUSTAL W algorithm within msa to conduct the alignment [47].

**Figure 6:**
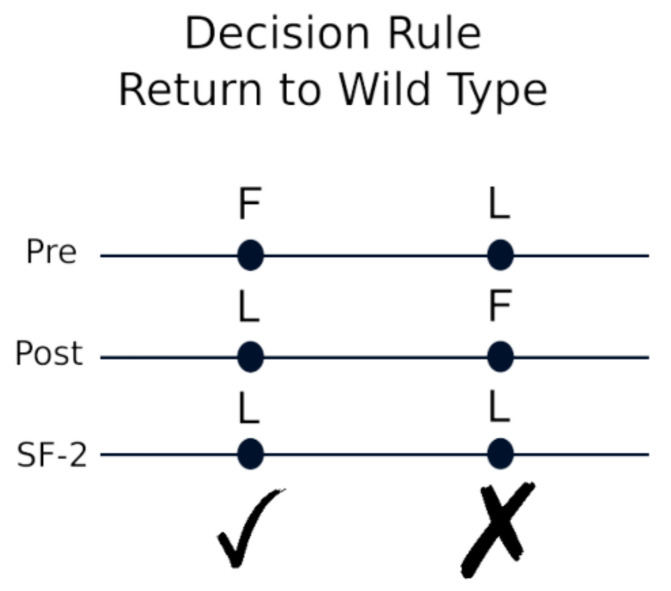
Schematic of decision rule for identifying codons returning to the wild type.

To determine the codons that were mutated pre-ATI and returned to the wild type post-ATI, we compared the amino acids at each codon. If the pre-ATI amino acid differed from the reference (wild-type) residue but returned to the wild type post-ATI, we then compared these cases to the IAS list mutations in the protease domain of the pol gene to examine patterns that might emerge. Figure 6 schematically illustrates the decision rule used to determine ‘hits’ to be examined.

## 5. Conclusions

These data indicate interesting features of the mutational patterns of HIV-1 in the gag gene in response to ART and its subsequent withdrawal which can be applied in research aiming to understand a key component of the viral replication process: the formation and maturation of new virions.

## Figures and Tables

**Table 1:** *Gag* cleavage sites from reference sequence SF-2.

Region		Pre-Cleavage					Post-Cleavage		
MA/CA	Codon	131	132	133	134	/	135	136	137
	Residue	S	Q	N	Y	/	P	I	V
CA/p2	Codon	362	363	364	365	/	366	367	368
	Residue	A	R	V	L	/	A	E	A
p2/NC	Codon	377	378	379	380	/	381	382	383
	Residue	A	N	I	M	/	M	Q	R
NC/p1	Codon	431	432	433	434	/	435	436	437
	Residue	R	Q	A	N	/	F	L	G
p1/p6	Codon	447	448	449	450	/	451	452	453
	Residue	P	G	N	F	/	L	Q	S

**Table 2:** Mutations per codon across gag regions.

Region	Length in Codons	Mutations Pre-ATI	Mutations Post-ATI	Difference in Mutations
Matrix	134	2.20	2.10	0.10
Capsid	231	1.07	1.03	0.04
p2	15	5.53	5.53	0.00
Nucleocapsid	54	2.37	1.87	0.50
p1	16	3.38	1.88	1.50
p6	113	2.42	1.44	0.98

**Table 3:** Mutations on the cleavage sites.

Patient	Codon	Pre-ATI	Post-ATI	SF-2	Revert to Wild Type
**MA/CA Region**					
5	134	F	F	Y	FALSE
10	134	F	F	Y	FALSE
34	134	F	Y	Y	TRUE
**CA/p2 Region**					
34	364	I	I	V	FALSE
52	364	I	I	V	FALSE
**p2/NC Region**					
3	377	T	T	N	FALSE
3	378	V	V	I	FALSE
3	382	K	K	R	FALSE
4	377	T	T	A	FALSE
4	379	V	V	I	FALSE
5	377	T	T	N	FALSE
5	382	K	K	R	FALSE
6	377	A	A	N	FALSE
8	378	V	V	I	FALSE
10	377	T	T	N	FALSE
10	378	V	V	I	FALSE
14	378	V	V	I	FALSE
15	377	T	T	N	FALSE
15	382	K	K	R	FALSE
19	377	A	A	N	FALSE
21	380	T	T	N	FALSE
22	377	T	T	N	FALSE
23	377	T	T	N	FALSE
26	377	T	T	N	FALSE
34	377	T	T	N	FALSE
35	377	T	A	N	FALSE
41	377	S	S	N	FALSE
44	377	T	T	N	FALSE
44	378	V	V	I	FALSE
46	377	T	T	N	FALSE
46	378	V	V	I	FALSE
51	377	A	A	N	FALSE
53	377	T	T	N	FALSE
56	378	T	T	N	FALSE
56	379	V	V	I	FALSE
**NC/p1 Region**					
10	433	V	V	A	FALSE
23	433	V	V	A	FALSE
35	437	K	G	G	TRUE
47	431	N	N	T	FALSE
47	435	V	A	A	TRUE
56	434	I	I	A	FALSE
**p1/p6 Region**					
3	453	N	N	S	FALSE
4	452	P	P	L	FALSE
15	451	P	P	L	FALSE
19	453	N	N	S	FALSE
35	448	R	G	G	TRUE
35	451	L	P	L	FALSE

**Table 4:** *Gag* mutations and associated protease mutations.

		*Gag* Mutations				Major Protease Mutations (Plasma) from IAS	
Codon	Sequence	Pre-ATI	Post-ATI	SF-2	*Gag* Region	Number Mutations	Mutations
47	24pre	Y		N	Matrix	3	M46L V82F I84V
47	24post		N	N	Matrix	0	No major PR mutations
76	43pre	K		R	Matrix	1	L90M
76	43post		R	R	Matrix	0	No major PR mutations
79	6pre	F		Y	Matrix	1	L90M
79	6post		Y	Y	Matrix	1	L90M
102	35pre	D		E	Matrix	2	G48V V82A
102	35post		E	E	Matrix	0	No major PR mutations
103	35pre	Q		K	Matrix	2	G48V V82A
103	35post		K	K	Matrix	0	No major PR mutations
111	34pre	C		S	Matrix	1	L90M
111	34post		S	S	Matrix	1	L90M
114	28pre	R		K	Matrix	3	M46I I84V L90M
114	28post		K	K	Matrix	0	No major PR mutations
121	48pre	D		A	Matrix	2	I84V L90M
121	48post		A	A	Matrix	2	I84V L90M
122	48pre	T		A	Matrix	2	I84V L90M
122	48post		A	A	Matrix	2	I84V L90M
134 *	34pre	F		Y	Matrix	1	L90M
134 *	34post		Y	Y	Matrix	1	L90M
149	28pre	L		I	Capsid	3	M46I I84V L90M
149	28post		I	I	Capsid	0	No major PR mutations
217	28pre	E		V	Capsid	3	M46I I84V L90M
217	28post		V	V	Capsid	0	No major PR mutations
243	47pre	S		T	Capsid	3	M46I I84V L90M
243	47post		T	T	Capsid	0	No major PR mutations
375	47pre	N		T	p2	3	M46I I84V L90M
375	47post		T	T	p2	0	No major PR mutations
386	28pre	K		R	Nucleocapsid	3	M46I I84V L90M
386	28post		R	R	Nucleocapsid	0	No major PR mutations
390	1pre	R		K	Nucleocapsid	3	M46L V82F I84V
390	1post		K	K	Nucleocapsid	0	No major PR mutations
420	52pre	K		R	Nucleocapsid	3	G48V V82A I84V
420	52post		R	R	Nucleocapsid	0	No major PR mutations
435 *	47pre	V		A	p1	3	M46I I84V L90M
435 *	47post		A	A	p1	0	No major PR mutations
451 *	35pre	P		L	p6	2	G48V V82A
451 *	35post		L	L	p6	0	No major PR mutations
455	28pre	L		P	p6	3	M46I I84V L90M
455	28post		P	P	p6	0	No major PR mutations
455	43pre	T		P	p6	1	L90M
455	43post		P	P	p6	0	No major PR mutations
457	47pre	L		P	p6	3	M46I I84V L90M
457	47post		P	P	p6	0	No major PR mutations
469	47pre	L		F	p6	3	M46I I84V L90M
469	47post		F	F	p6	0	No major PR mutations
472	36pre	A		T	p6	3	M46I V82A L90M
472	3post		T	T	p6	0	No major PR mutations
476	8pre	I		T	p6	2	M46I I84V
476	8post		T	T	p6	0	No major PR mutations

An asterisk (*) in the list of codons means that this codon falls in a cleavage site.

## Data Availability

We have deposited all data and analyses at https://github.com/jameshunterbr/ATI accessed on 8 October 2021, with open access to the public.

## Supplementary Materials

The following supporting information can be downloaded at: https://www.mdpi.com/article/10.3390/pathogens11050534/s1, Table S1: Sample size in parallel studies, Table S2: Retroviral use before interruption, Table S3: *Gag* mutations and associated protease mutations, Table S4: *Gag* mutations and minor associated protease mutations.
