# Supplementary material for: Antiretroviral Drug-Resistance Mutations on the Gag Gene: Mutation Dynamics during Analytic Treatment Interruption among Individuals Experiencing Virologic Failure
Source: Pathogens. 2022 May 3;11(5):534. doi: 10.3390/pathogens11050534 (PMC9145614; doi:10.3390/pathogens11050534)
Supplement: Supplementary file 1 [file pathogens-11-00534-s001.zip › SUPP/S3_Table_gag.pdf]

		gag Mutations			*Major Protease Mutations (plasma) from IAS*	
**Codon**	**Sequence**	**Pre-ATI**	**Post-ATI**	***gag* Region**	**# Mutations**	**Mutations**
47	24pre	Y		matrix	3	M46L V82F I84V
47	24post		N	matrix	0	No major PR mutations
49	10pre	G		matrix	3	M46I I84V L90M
49	10post		A	matrix	3	M46I I84V L90M
55	35pre	G		matrix	2	G48V V82A
55	35post		E	matrix	0	No major PR mutations
61	48pre	I		matrix	2	I84V L90M
61	48post		L	matrix	2	I84V L90M
62	48pre	E		matrix	2	I84V L90M
62	48post		G	matrix	2	I84V L90M
67	48pre	A		matrix	2	I84V L90M
67	48post		S	matrix	2	I84V L90M
68	48pre	I		matrix	2	I84V L90M
68	48post		L	matrix	2	I84V L90M
69	48pre	K		matrix	2	I84V L90M
69	48post		Q	matrix	2	I84V L90M
76	35pre	K		matrix	2	G48V V82A
76	35post		R	matrix	0	No major PR mutations
76	43pre	K		matrix	1	L90M
76	43post		R	matrix	0	No major PR mutations
76	48pre	K		matrix	2	I84V L90M
76	48post		R	matrix	2	I84V L90M
79	6pre	F		matrix	1	L90M
79	6post		Y	matrix	1	L90M
79	48pre	F		matrix	2	I84V L90M
79	48post		Y	matrix	2	I84V L90M
84	35pre	V		matrix	2	G48V V82A
84	35post		T	matrix	0	No major PR mutations
91	35pre	K		matrix	2	G48V V82A
91	35post		R	matrix	0	No major PR mutations
91	47pre	K		matrix	3	M46I I84V L90M
91	47post		R	matrix	0	No major PR mutations
102	35pre	D		matrix	2	G48V V82A

Codon	Sequence	*gag Mutations* Pre-ATI	Post-ATI	*gag* Region	*Major Protease Mutations (plasma) from IAS* # Mutations	Mutations
102	35post		E	matrix	0	No major PR mutations
102	46pre	E		matrix	3	G48V V82A L90M
102	46post		D	matrix	0	No major PR mutations
102	47pre	D		matrix	3	M46I I84V L90M
102	47post		E	matrix	0	No major PR mutations
102	48pre	D		matrix	2	I84V L90M
102	48post		E	matrix	2	I84V L90M
103	35pre	Q		matrix	2	G48V V82A
103	35post		K	matrix	0	No major PR mutations
110	46pre	K		matrix	3	G48V V82A L90M
110	46post		E	matrix	0	No major PR mutations
111	34pre	C		matrix	1	L90M
111	34post		S	matrix	1	L90M
114	28pre	R		matrix	3	M46I I84V L90M
114	28post		K	matrix	0	No major PR mutations
114	35pre	Q		matrix	2	G48V V82A
114	35post		K	matrix	0	No major PR mutations
120	48pre	T		matrix	2	I84V L90M
120	48post		A	matrix	2	I84V L90M
121	35pre	G		matrix	2	G48V V82A
121	35post		A	matrix	0	No major PR mutations
121	47pre	D		matrix	3	M46I I84V L90M
121	47post		A	matrix	0	No major PR mutations
121	48pre	D		matrix	2	I84V L90M
121	48post		A	matrix	2	I84V L90M
122	35pre	T		matrix	2	G48V V82A
122	35post		A	matrix	0	No major PR mutations
122	47pre	T		matrix	3	M46I I84V L90M
122	47post		A	matrix	0	No major PR mutations
122	48pre	T		matrix	2	I84V L90M
122	48post		A	matrix	2	I84V L90M

		gag Mutations			Major Protease Mutations (plasma) from IAS	
**Codon**	**Sequence**	**Pre-ATI**	**Post-ATI**	***gag* Region**	**# Mutations**	**Mutations**
124	35pre	N		matrix	2	G48V V82A
124	35post		T	matrix	0	No major PR mutations
125	35pre	S		matrix	2	G48V V82A
125	35post		G	matrix	0	No major PR mutations
134*	34pre	F		matrix	1	L90M
134*	34post		Y	matrix	1	L90M
149	28pre	L		capsid	3	M46I I84V L90M
149	28post		I	capsid	0	No major PR mutations
212	44pre	A		capsid	3	M46I I84V L90M
212	44post		V	capsid	0	No major PR mutations
217	28pre	E		capsid	3	M46I I84V L90M
217	28post		V	capsid	0	No major PR mutations
217	35pre	L		capsid	2	G48V V82A
217	35post		V	capsid	0	No major PR mutations
217	48pre	L		capsid	2	I84V L90M
217	48post		V	capsid	2	I84V L90M
219	47pre	L		capsid	3	M46I I84V L90M
219	47post		V	capsid	0	No major PR mutations
222	47pre	I		capsid	3	M46I I84V L90M
222	47post		V	capsid	0	No major PR mutations
243	47pre	S		capsid	3	M46I I84V L90M
243	47post		T	capsid	0	No major PR mutations
251	4pre	A		capsid	2	G48V V82A
251	4post		P	capsid	0	No major PR mutations
254	35pre	H		capsid	2	G48V V82A
254	35post		N	capsid	0	No major PR mutations
256	47pre	S		capsid	3	M46I I84V L90M
256	47post		N	capsid	0	No major PR mutations
273	21pre	L		capsid	3	M46L V82F L90M
273	21post		M	capsid	0	No major PR

Codon	Sequence	*gag* Mutations Pre-ATI	Post-ATI	*gag* Region	***Major Protease Mutations (plasma) from IAS*** # Mutations	Mutations
						mutations
282	48pre	V		capsid	2	I84V L90M
282	48post		T	capsid	2	I84V L90M
288	35pre	K		capsid	2	G48V V82A
288	35post		R	capsid	0	No major PR mutations
312	35pre	T		capsid	2	G48V V82A
312	35post		S	capsid	0	No major PR mutations
314	35pre	E		capsid	2	G48V V82A
314	35post		D	capsid	0	No major PR mutations
316	47pre	E		capsid	3	M46I I84V L90M
316	47post		D	capsid	0	No major PR mutations
350	48pre	S		capsid	2	I84V L90M
350	48post		T	capsid	2	I84V L90M
373	23pre	T		p2	3	M46L V82F I84V
373	23post		N	p2	3	M46L V82F I84V
375	35pre	T		p2	2	G48V V82A
375	35post		P	p2	0	No major PR mutations
375	47pre	N		p2	3	M46I I84V L90M
375	47post		T	p2	0	No major PR mutations
376	35pre	N		p2	2	G48V V82A
376	35post		A	p2	0	No major PR mutations
377*	35pre	A		p2	2	G48V V82A
377*	35post		N	p2	0	No major PR mutations
379*	47pre	T		p2	3	M46I I84V L90M
379*	47post		N	p2	0	No major PR mutations
386	28pre	K		nucleocapsid	3	M46I I84V L90M
386	28post		R	nucleocapsid	0	No major PR mutations
386	44pre	R		nucleocapsid	3	M46I I84V L90M
386	44post		K	nucleocapsid	0	No major PR mutations
387	47pre	P		nucleocapsid	3	M46I I84V L90M
387	47post		F	nucleocapsid	0	No major PR

Codon	Sequence	gag Mutations: Pre-ATI	Post-ATI	gag Region	Major Protease Mutations (plasma) from IAS: # Mutations	Mutations
						mutations
388	47pre	G		nucleocapsid	3	M46I I84V L90M
388	47post		R	nucleocapsid	0	No major PR mutations
390	1pre	R		nucleocapsid	3	M46L V82F I84V
390	1post		K	nucleocapsid	0	No major PR mutations
391	48pre	I		nucleocapsid	2	I84V L90M
391	48post		T	nucleocapsid	2	I84V L90M
392	48pre	I		nucleocapsid	2	I84V L90M
392	48post		V	nucleocapsid	2	I84V L90M
393	47pre	I		nucleocapsid	3	M46I I84V L90M
393	47post		T	nucleocapsid	0	No major PR mutations
402	47pre	V		nucleocapsid	3	M46I I84V L90M
402	47post		E	nucleocapsid	0	No major PR mutations
417	48pre	K		nucleocapsid	2	I84V L90M
417	48post		R	nucleocapsid	2	I84V L90M
419	47pre	K		nucleocapsid	3	M46I I84V L90M
419	47post		R	nucleocapsid	0	No major PR mutations
420	35pre	K		nucleocapsid	2	G48V V82A
420	35post		R	nucleocapsid	0	No major PR mutations
420	48pre	K		nucleocapsid	2	I84V L90M
420	48post		R	nucleocapsid	2	I84V L90M
420	52pre	K		nucleocapsid	3	G48V V82A I84V
420	52post		R	nucleocapsid	0	No major PR mutations
422	47pre	K		nucleocapsid	3	M46I I84V L90M
422	47post		R	nucleocapsid	0	No major PR mutations
429	35pre	S		nucleocapsid	2	G48V V82A
429	35post		T	nucleocapsid	0	No major PR mutations
431*	47pre	N		nucleocapsid	3	M46I I84V L90M
431*	47post		T	nucleocapsid	0	No major PR mutations
435*	47pre	V		p1	3	M46I I84V L90M
435*	47post		A	p1	0	No major PR mutations

Codon	Sequence	*gag* Mutations			*Major Protease Mutations (plasma) from IAS*	
		Pre-ATI	Post-ATI	*gag* Region	# Mutations	Mutations
443	35pre	H		p1	2	G48V V82A
443	35post		Y	p1	0	No major PR mutations
443	36pre	Q		p1	3	M46I V82A L90M
443	36post		H	p1	0	No major PR mutations
443	48pre	R		p1	2	I84V L90M
443	48post		Y	p1	2	I84V L90M
445	47pre	S		p1	3	M46I I84V L90M
445	47post		Y	p1	0	No major PR mutations
445	48pre	E		p1	2	I84V L90M
445	48post		G	p1	2	I84V L90M
451*	35pre	P		p6	2	G48V V82A
451*	35post		L	p6	0	No major PR mutations
455	28pre	L		p6	3	M46I I84V L90M
455	28post		P	p6	0	No major PR mutations
455	43pre	T		p6	1	L90M
455	43post		P	p6	0	No major PR mutations
457	47pre	L		p6	3	M46I I84V L90M
457	47post		P	p6	0	No major PR mutations
458	34pre	T		p6	1	L90M
458	34post		S	p6	1	L90M
458	35pre	S		p6	2	G48V V82A
458	35post		T	p6	0	No major PR mutations
469	47pre	L		p6	3	M46I I84V L90M
469	47post		F	p6	0	No major PR mutations
471	35pre	T		p6	2	G48V V82A
471	35post		K	p6	0	No major PR mutations
472	36pre	A		p6	3	M46I V82A L90M
472	36post		T	p6	0	No major PR mutations
473	47pre	T		p6	3	M46I I84V L90M
473	47post		K	p6	0	No major PR mutations

Codon	Sequence	*gag* Mutations: Pre-ATI	Post-ATI	*gag* Region	*Major Protease Mutations (plasma) from IAS*: # Mutations	Mutations
476	8pre	I		p6	2	M46I I84V
476	8post		T	p6	0	No major PR mutations
478	48pre	Q		p6	2	I84V L90M
478	48post		E	p6	2	I84V L90M
479	48pre	T		p6	2	I84V L90M
479	48post		K	p6	2	I84V L90M
483	1pre	K		p6	3	M46L V82F I84V
483	1post		T	p6	0	No major PR mutations
489	35pre	A		p6	2	G48V V82A
489	35post		T	p6	0	No major PR mutations
491	47pre	A		p6	3	M46I I84V L90M
491	47post		T	p6	0	No major PR mutations
497	48pre	A		p6	2	I84V L90M
497	48post		T	p6	2	I84V L90M
