# Supplementary material for: Antiretroviral Drug-Resistance Mutations on the Gag Gene: Mutation Dynamics during Analytic Treatment Interruption among Individuals Experiencing Virologic Failure
Source: Pathogens. 2022 May 3;11(5):534. doi: 10.3390/pathogens11050534 (PMC9145614; doi:10.3390/pathogens11050534)
Supplement: Supplementary file 1 [file pathogens-11-00534-s001.zip › SUPP/S4_Table_gag.pdf]

Codon	Sequence	Gag Mutations pre-STI	post-STI	SF-2	*gag* Region		Minor Protease Mutations (plasma) from IAS # Mutations	Mutations
47	24pre	Y		N	matrix		8	L10V K20R L24I M36I I54V L63P A71T G73S
47	24post		N	N	matrix		0	No minor PR mutations
76	43pre	K		R	matrix		4	L10I M36I L63P A71I
76	43post		R	R	matrix		4	L10I M36I L63P A71T
79	6pre	F		Y	matrix		3	M36I L63P A71T
79	6post		Y	Y	matrix		4	K20R M36I L63P A71T
102	35pre	D		E	matrix		4	L10V K20R M36I I54V
102	35post		E	E	matrix		2	L10V M36I
103	35pre	Q		K	matrix		4	L10V K20R M36I I54V
103	35post		K	K	matrix		2	L10V M36I
111	34pre	C		S	matrix		6	L10I/V K20M L63P A71T G73S V77I
111	34post		S	S	matrix		4	L10V L63P A71T V77I
114	28pre	R		K	matrix		5	L10I L63P A71V G73S V77I
114	28post		K	K	matrix		3	L63P A71T V77I
121	48pre	D		A	matrix		3	M36I I54V L63P
121	48post		A	A	matrix		3	M36I I54V L63P
122	48pre	T		A	matrix		3	M36I I54V L63P
122	48post		A	A	matrix		3	M36I I54V L63P
134*	34pre	F		Y	matrix		6	L10I/V K20M L63P A71T G73S V77I
134*	34post		Y	Y	matrix		4	L10V L63P A71T V77I
149	28pre	L		I	capsid		5	L10I L63P A71V G73S V77I
149	28post		I	I	capsid		3	L63P A71T V77I
217	28pre	E		V	capsid		5	L10I L63P A71V G73S V77I
217	28post		V	V	capsid		3	L63P A71T V77I
243	47pre	S		T	capsid		6	L10I M36I F53L I54V L63V/A/I/T A71V
243	47post		T	T	capsid		1	L10I
375	47pre	N		T	p2		6	L10I M36I F53L I54V L63V/A/I/T A71V
375	47post		T	T	p2		1	L10I
386	28pre	K		R	nucleocapsid		5	L10I L63P A71V G73S V77I
386	28post		R	R	nucleocapsid		3	L63P A71T V77I
390	1pre	R		K	nucleocapsid		5	L10V K20R M36I I54V A71T
390	1post		K	K	nucleocapsid		2	A71T V77I
420	52pre	K		R	nucleocapsid		5	L10I K20R L24I L33F I54V
420	52post		R	R	nucleocapsid		1	V77I
435*	47pre	V		A	p1		6	L10I M36I F53L I54V L63V/A/I/T A71V
435*	47post		A	A	p1		1	L10I
451*	35pre	P		L	p6		4	L10V K20R M36I I54V
451*	35post		L	L	p6		2	L10V M36I
455	28pre	L		P	p6		5	L10I L63P A71V G73S V77I

Codon	Sequence	*Gag Mutations*					*Minor Protease Mutations (plasma) from IAS*	
		pre-STI	post-STI	SF-2	*gag* Region		# Mutations	Mutations
455	28post		P	P	p6		3	L63P A71T V77I
455	43pre	T		P	p6		4	L10I M36I L63P A71I
455	43post		P	P	p6		4	L10I M36I L63P A71T
457	47pre	L		P	p6		6	L10I M36I F53L I54V L63V/A/I/T A71V
457	47post		P	P	p6		1	L10I
469	47pre	L		F	p6		6	L10I M36I F53L I54V L63V/A/I/T A71V
469	47post		F	F	p6		1	L10I
472	36pre	A		T	p6		5	L10I I54V L63P A71V N88D
472	3post		T	T	p6		1	L63P
476	8pre	I		T	p6		5	L10I F53L A71V V77I N88D
476	8post		T	T	p6		1	V77I
